# Pannexin-1 Channels Are Essential for Mast Cell Degranulation Triggered During Type I Hypersensitivity Reactions

**DOI:** 10.3389/fimmu.2019.02703

**Published:** 2019-11-29

**Authors:** Paloma A. Harcha, Ximena López, Pablo J. Sáez, Paola Fernández, Iván Barría, Agustín D. Martínez, Juan C. Sáez

**Affiliations:** ^1^Departamento de Fisiología, Facultad de Ciencias Biológicas, Pontificia Universidad Católica de Chile, Santiago, Chile; ^2^Facultad de Ciencias, Instituto de Neurociencias and Centro Interdisciplinario de Neurociencias de Valparaíso, Universidad de Valparaíso, Valparaíso, Chile; ^3^Institut Curie, PSL Research University, CNRS, UMR 144, Paris, France; ^4^Institut Pierre-Gilles de Gennes, PSL Research University, Paris, France

**Keywords:** inflammation, immune cells, ovalbumin, pannexon, degranulation, histamine, ATP

## Abstract

Mast cells (MCs) release pro-inflammatory mediators through a process called degranulation response. The latter may be induced by several conditions, including antigen recognition through immunoglobulin E (IgE) or “cross-linking,” classically associated with Type I hypersensitivity reactions. Early in this reaction, Ca^2+^ influx and subsequent increase of intracellular free Ca^2+^ concentration are essential for MC degranulation. Several membrane channels that mediate Ca^2+^ influx have been proposed, but their role remains elusive. Here, we evaluated the possible contribution of pannexin-1 channels (Panx1 Chs), well-known as ATP-releasing channels, in the increase of intracellular Ca^2+^ triggered during cross-linking reaction of MCs. The contribution of Panx1 Chs in the degranulation response was evaluated in MCs from wild type (WT) and Panx1 knock out (Panx1^−/−^) mice after anti-ovalbumin (OVA) IgE sensitization. Notably, the degranulation response (toluidine blue and histamine release) was absent in Panx1^−/−^ MCs. Moreover, WT MCs showed a rapid and transient increase in Ca^2+^ signal followed by a sustained increase after antigen stimulation. However, the sustained increase in Ca^2+^ signal triggered by OVA was absent in Panx1^−/−^ MCs. Furthermore, OVA stimulation increased the membrane permeability assessed by dye uptake, a prevented response by Panx1 Ch but not by connexin hemichannel blockers and without effect on Panx1^−/−^ MCs. Interestingly, the increase in membrane permeability of WT MCs was also prevented by suramin, a P2 purinergic inhibitor, suggesting that Panx1 Chs act as ATP-releasing channels impermeable to Ca^2+^. Accordingly, stimulation with exogenous ATP restored the degranulation response and sustained increase in Ca^2+^ signal of OVA stimulated Panx1^−/−^ MCs. Moreover, opening of Panx1 Chs in Panx1 transfected HeLa cells increased dye uptake and ATP release but did not promote Ca^2+^ influx, confirming that Panx1 Chs permeable to ATP are not permeable to Ca^2+^. These data strongly suggest that during antigen recognition, Panx1 Chs contribute to the sustained Ca^2+^ signal increase via release of ATP that activates P2 receptors, playing a critical role in the sequential events that leads to degranulation response during Type I hypersensitivity reactions.

## Introduction

Mast cells (MCs) originate from hematopoietic precursors, which migrate through the bloodstream and differentiate in diverse tissues. These areas include surfaces normally exposed to pathogens such as skin, gastrointestinal, and airway epithelium (1, 2). Upon activation, MCs rapidly respond by releasing pro-inflammatory mediators that recruit several effector cells, quickly triggering a local inflammatory response characterized by vasodilatation, congestion, and edema.

The best-described MC responses occurs during Type I hypersensitivity reactions, which are manifested in various allergic diseases, such as allergic asthma, most types of sinusitis, allergic rhinitis, and food allergies. Under these pathological conditions, the host produces large amounts of plasmatic immunoglobulin E (IgE) (3–6) that binds and stabilizes their receptors (FcεRI) (7), coating and sensitizing MCs to specific antigens. IgE bound to FcεRI up-regulates FcεRI expression on MCs via stabilization and accumulation of FcεRI (8). After sensitization, a subsequent antigen exposure is recognized through the IgE–FcεRI interaction, or cross-linking, leading to a cascade of intracellular events that result in rapid degranulation and synthesis of lipid-derived mediators and cytokines (9). In the particular case of the degranulation response induced by the IgE–FcεRI interaction, MCs within few seconds release to the extracellular milieu pre-formed mediators such as TNF-α, ATP, and histamine, amplifying an inflammatory response (10). The mechanism that allows release of the granules content includes influx of calcium ions (Ca^2+^) (11–15).

Since Ca^2+^ influx is a common mechanism induced by activation of different antigen immunoreceptors found in B and T cells (16), and as ATP released through pannexin-1 channels (Panx1 Chs) has been related to TCR activation (17, 18), we decided to evaluate whether Panx1 Chs expressed by MCs contribute to the Ca^2+^ signal triggered by IgE-antigen cross-linking. Panx1 forms non-selective membrane channels ubiquitously expressed in vertebrates (19). They are permeable to ion, small molecules such as ATP, as well as fluorescent permeability tracers (20). However, their permeability varies according to the gating mechanism that activates them. While Panx1 Chs opened by membrane depolarization present low unitary conductance and are selectively permeable to chloride anion (Cl^−^), Panx1 Chs opened by high extracellular K^+^ are non-selective channels and present higher unitary conductance, allowing the release of ATP (20).

Since we have previously shown that MCs express Panx1 Chs (21), we decided to study their possible role in Type I hypersensitivity reaction. For this purpose, we first compared the degranulation response on bone marrow MCs derived from wild type (WT) and Panx 1 knock-out (Panx1^−/−^) mice after 6 h coating with anti-ovalbumin IgE. The degranulation response was absent in Panx1^−/−^ MCs, consistent with lack of persistent phase of the Ca^2+^ signal in these cells. In MCs from WT mice, the antigen-induced dye uptake was prevented by Panx1 Ch and P2 receptor blockers and was absent in Panx1^−/−^MCs. Moreover, stimulation with exogenous ATP restored degranulation, dye uptake, and persistent phase of the Ca^2+^ signal in Panx1^−/−^ MCs. In addition, the opening of Panx1 Chs expressed in HeLa cells induced ATP release but did not affect the intracellular Ca^2+^ signal, suggesting that Panx1 Chs are permeable to ATP but not to Ca^2+^. Thus, we propose that in MC activated by cross-linking Panx1 Chs contribute to the intracellular Ca^2+^ signal increase via ATP release, which subsequently activate P2 receptors, causing a persistent increase in Ca^2+^ signal required for degranulation.

## Materials and Methods

### Chemicals

RPMI 1640, DMEM, and IMDM culture mediums; penicillin; streptomycin; and fetal bovine serum (FBS) were obtained from Gibco®Invitrogen (Carlsbad, CA, USA). Albumin from chicken egg white (OVA), 2-mercaptoethanol, Probenecid (Pbc), carbenoxolone (Cbx), suramin, ethidium (Etd) bromide, and 4′,6-diamidino-2-phenylindole (DAPI) were purchased from Sigma Chemical (St. Louis, MO, USA). ^10^Panx1 (WRQAAFVDSY) and Gap26 (FSVYWAQADR) mimetic peptides were obtained from SBSBIO (Beijing, China). Mouse monoclonal IgE antibody against OVA was purchased from AbD Serotec (Kidlington, UK).

### Isolation, Differentiation, and Sensitization of MCs

All animal procedures were performed in accordance with the approval of the Committee of Bioethics and Biosecurity from the Pontificia Universidad Católica de Chile.

MCs were obtained from 2-month C57Bl/6 (WT) and C57Bl/6-Panx1^−/−^ (Panx1^−/−^) male mice. Panx1^−/−^ mice, generated as described (22, 23), were kindly donated by Dr. Hanna Monyer (University of Heilderberg, Germany).

MCs were prepared as previously described (24). In brief, bone marrow precursor cells from tibia and femur bones were flushed out to RPMI 1640 media, supplemented with 20% WEHI conditioned media [from cultured WEHI-3 cells (TIB-68, ATCC)], 10% FBS, 50 μM 2-mercaptoethanol, and 100 units/ml of penicillin and streptomycin. Then, precursor cells were plated on non-activated T25 plastic flasks and media was changed every 4 days. After 4 weeks, cell suspensions were used. For sensitization, 1 × 10^6^ MCs were incubated with 1 μg/ml of monoclonal IgE against OVA for 6 h at 37°C.

### HeLa Cells Culture

Parental HeLa cells (HeLa-Parental) and HeLa cells stably transfected with mouse connexin43 (HeLa-Cx43) or mouse Panx1 (HeLa-Panx1) were kindly donated by Dr. Klaus Willecke (Limus Institute, Bonn University, Germany) and Dr. Feliksas F. Bukaukas (Department of Neuroscience, Albert Einstein College of Medicine, Bronx, NY), respectively. Cells were cultured in DMEM media supplemented with 10% FBS and 50 U/ml penicillin and streptomycin, at 37°C in a 5% CO_2_/95% O_2_. HeLa-Cx43 cell and 100% humidity atmosphere. HeLa-Cx43 cells transfection were maintained with 0.01 mg/ml puromycin while HeLa-Panx1 cells were maintained with 1 mg/ml G418 (Invitrogen).

### Time-Lapse Fluorescence Imaging

For dye uptake assays, sensitized MCs were first seeded on 0.05% w/v poly-L-lysine coated coverslips and then placed on a recording Locke's solution (154 mM NaCl, 5.4 mM KCl, 2.3 mM CaCl_2_, 1 mM MgCl_2_, 5 mM glucose, 5 mM HEPES, at pH 7.42) containing 5 μM Etd or 5 μM DAPI, as we have previously described (23) Etd and DAPI are permeability tracers used to evaluate the functional state of Panx1 Chs and Cx43 hemichannels (HCs). Fluorescence intensity images were recorded in selected cells (ROIs, regions of interest) with a NIKON Eclipse Ti inverted microscope (Japan) every 30 s during 20 min using a Q Imaging model Retiga 13001 fast-cooled monochromatic digital camera (12-bit) (Qimaging, Burnaby, BC, Canada) to detect changes in fluorescence intensity in every condition. Metafluor software (version 6.2R5, Universal Imaging Co., Downingtown, PA, USA) was used for off-line image analysis. Then, changes in fluorescence intensity slope (expressed as AU/min) were compared after regression lines fitted to points using Microsoft Excel (Seattle, WA), and slope values were normalized using ImageJ 1.64r software (Bethesda, MD, USA) and plotted with GraphPad Prism 5 (San Diego, California, USA).

### Degranulation Assays

For histamine quantification, we used a fluorometric assay based on its stable condensation with O-phthalaldehyde (OPT) in an alkaline medium (25). Briefly, sensitized 25 × 10^4^ cells were placed in 200 μl of recording solution for antigen stimulation. Then, cells were centrifuged and supernatants were collected and exposed to 10 μl OPT (7.5 μM) and 40 μl NaOH (1 mM) for 4 min. After stabilization with 20 μl of HCl (3 mM), fluorescence at 360 nm in a conventional spectrophotometer was quantified.

Being an alkaline dye, toluidine blue (TB) binds to proteoglycans present in MC granules and is released during the degranulation response (26). Thus, TB release was used as an indicator of MC degranulation (24). After MC adhesion on poly-L-lysine-coated coverslips, cells were incubated with 1.8 mM TB for 10 min at room temperature, washed three times, and immersed in recording solution for time-lapse imaging. Bright-field images were captured with a NIKON Eclipse Ti inverted microscope (Japan) every 30 s for 15 min. Blue intensity loss was quantified with ImageJ 1.64r program (Bethesda, MD/Meriland, USA) and plotted with GraphPad Prism 5 (San Diego, California, USA) software.

### Intracellular Calcium (Ca^2+^) Signal

After adhesion on poly-L-lysine glass coverslips, MCs and transfected HeLa cells were loaded with 5 μM Fura-2AM RPMI 1640 medium for 30 min at 37°C. After washing, cells were bathed with recording Locke's solution and fluorescence images were captured every 3 s. Experimental protocol for imaging involved data acquisition of light emission at 510 nm due to excitation at 340 and 380 nm. The ratio was obtained by dividing the emission fluorescence image value obtained at 340 nm by that recorded at 380-nm excitation on a pixel-by-pixel base (Ca^2+^ signal = F340/F380 nm). Images and ratio quantification were performed in the NIKON Eclipse Ti inverted microscope and imaging Nis Elements software (Japan) while the data were plotted with GraphPad Prism 5 (San Diego, California, USA) software.

### Measurement of ATP Release

Extracellular ATP was quantified using the luciferin-luciferase ATP determination assay kit (Invitrogen, Eugene, OR). Briefly, 10,000 cells were placed on p35 plastic plaques (NUNC) in complete DMEM media. Twenty-four hours later, cells were carefully washed twice with 1 ml of buffer solution each time and then mixed with 1 ml of saline solution at pH 7.4 or 8.5 was added. After 10 min of incubation, two samples of 10 μl each were collected and mixed with 90 μl of ATP-mix solution and placed on a plastic 96-well plate, following kit manufacture recommendations. Then, luminescence was determined using a Luminescence Spectrometer LS50B (Perkin-Elmer, Massachusetts, USA).

### Statistics

A minimal animal sample was defined after Mead's resource equation (1988) with no stratification and 20 for degrees of freedom. All data in this work are presented as mean ± SEM. Since data were parametric according to Shapiro–Wilk normality test, each condition was compared with its respective control using one-way ANOVA test, and significance was determined with *a posteriori* Tukey test using GraphPad Prism 7 software.

## Results

### Panx1 Is Essential for MC Degranulation Induced by Antigen Cross-Linking Recognition

During antigen cross-linking recognition reaction, MCs release several preformed mediators present in cytoplasmic granules, such as histamine. Here, we measured soluble histamine in cell supernatants as an indicator for MC degranulation.

**Figure 1:**
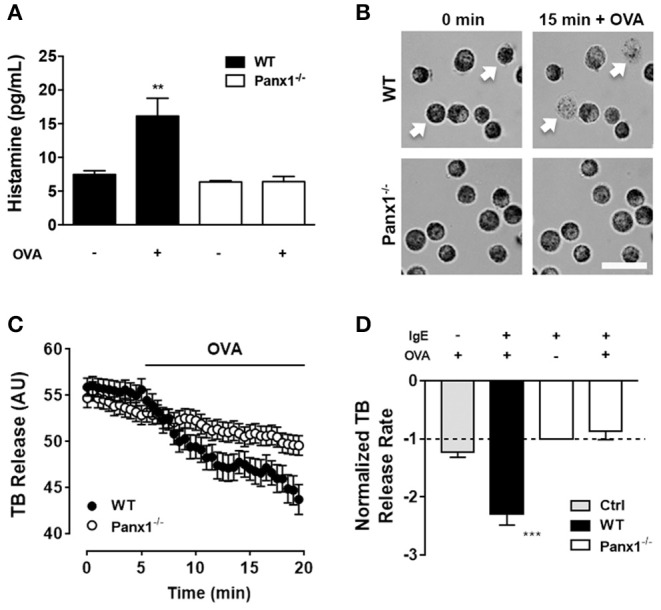
Degranulation induced by OVA cross-linked recognition depends on Panx1 expression. **(A)** Extracellular histamine concentration in IgE-sensitized mast cells (MCs) cultured under basal conditions and after 20 min stimulation with 10 μM OVA in wild type (WT, black) and Panx1^−/−^ mast cells (MCs) (white). **(B)** Representative images of toluidine blue (TB)-loaded WT (upper panels) and Panx1^−/−^ MCs (lower panels) before and after 15 min treatment with 10 μM OVA. White bar: 20 μm. **(C)** Time-lapse measurements of blue intensity recorded in WT (black circles) and Panx1^−/−^ (white circles) MCs before and after treatment with OVA (black trace). **(D)** Normalized blue intensity loss rate induced by OVA with respect to basal conditions in control non-sensitized MCs (Ctrl, gray), WT (black), and Panx1^−/−^ MCs (white). Each point corresponds to the mean ± SEM, *n* = 4, between 30 and 50 cells were recorded in each experiment, ***p < 0.0005; **p < 0.005.

Soluble histamine quantification was performed under control conditions and after 20 min of OVA exposure to IgE-sensitized WT and Panx1^−/−^ MCs. In WT MCs, OVA exposure increased to about double the amount of extracellular histamine [16 ± 3 pg after OVA stimulation, vs. 8 ± 1 pg under basal condition (*n* = 4), ^**^p < 0.005]. However, no increase was observed in IgE-sensitized Panx1^−/−^ MCs exposed to OVA [6 ± 1 after OVA stimulation vs. 6± 2 under basal conditions (*n* = 4), *p* > 0.05] (Figure 1A), suggesting that Panx1 is required for histamine release after OVA recognition in sensitized MCs.

Additionally, we evaluated MC degranulation in time-lapse experiments. For this purpose, proteoglycan containing granules were loaded with TB, and blue intensity loss was quantified under different conditions. Basal TB loss from WT and Panx1^−/−^ IgE-sensitized MCs was recorded during 5 min and then cells were exposed to 10 μM OVA (Figure 1B). Quantification of normalized rates of dye loss (slope) under basal conditions (first 5 min) and after OVA treatment (between 5 and 20 min of recording) of WT and Panx1^−/−^ sensitized MCs was plotted (Figure 1C). Only in Panx1-expressing sensitized MCs did TB intensity loss rate increase significantly (about 2.3 times) after OVA stimulation (*n* = 3, ^***^p < 0.0005), supporting the interpretation that Panx1 is crucial for MC degranulation after OVA cross-linking recognition. In control MCs (not sensitized, Ctrl), exposure to OVA did not induce significant change in TB release (Figure 1D), suggesting that IgE–FcεRI interaction plays a critical role in OVA-induced MC degranulation.

### The Ca^2+^ Signal Plateau After Antigen Cross-Linking Recognition Is Not Elicited by Panx1^−/−^ MCs

**Figure 2:**
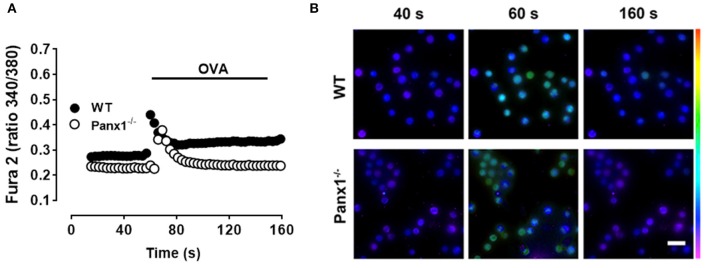
The OVA-induced Ca^2+^ signal of Panx1^−/−^ mast cells is partially impaired. **(A)** Representative intracellular Ca^2+^ signal in WT (black) and Panx1^−/−^ (white) mast cells (MCs). In Fura-2-loaded sensitized MCs, the Ca^2+^ signal was first recorded under basal conditions during 50 s and then were exposed to 10 μM OVA (black trace). Each value corresponds to the mean ± SEM of Ca^2+^ signal recorded in 50 cells. A total of five independent experiments were performed. **(B)** Fields of representative Fura-2 fluorescence before (40 s), during (60 s), and after (160 s) OVA exposure of WT and Panx1^−/−^ MCs treated as in **(A)**. Scale bar, 20 μm.

Since IgE-mediated antigen recognition promotes a Ca^2+^ signal that is essential for MC degranulation (27), we compared the Ca^2+^ signal response induced by OVA in both IgE-sensitized WT and Panx1^−/−^ MCs. Figure 2A illustrates representative images under resting conditions (40 s), after acute stimulation (60 s), and after 100 s of antigen recognition (160 s) in both WT (upper panels) and Panx1^−/−^ MCs (lower panels) loaded with Fura-2. Antigen recognition promoted first a rapid and transient phase followed by a sustained Ca^2+^ signal increase higher than the basal values (Figure 2B), as previously shown by others (28). Moreover, in IgE-sensitized Panx1^−/−^ MCs exposed to OVA, the rapid and transient phase was evident but values recovered to the basal conditions and the subsequent plateau phase elicited by WT MCs was absent (Figure 2B), suggesting that Panx1 participates in the sustained Ca^2+^ influx phase induced by IgE-antigen recognition.

### Antigen Recognition Through Cross-Linking Induces Panx1-Dependent Dye Uptake in BMMCs

**Figure 3:**
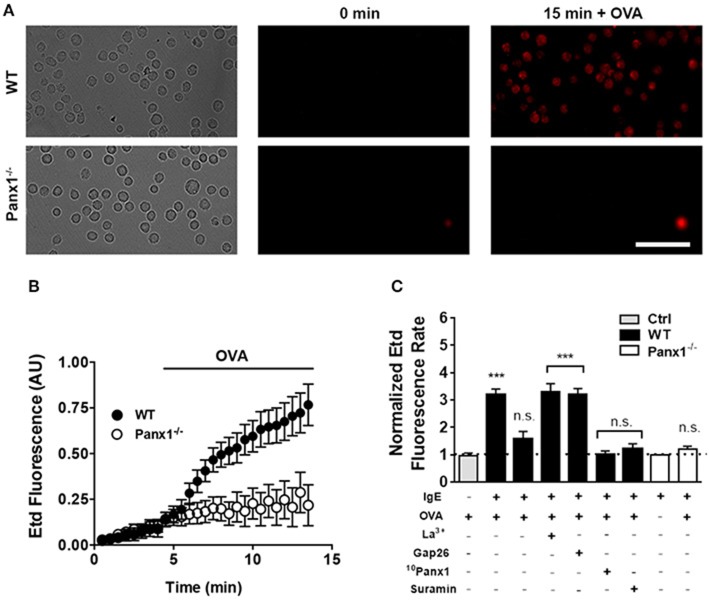
OVA recognition promotes Panx1-dependent membrane permeability changes in sensitized mast cells. The membrane permeability of mast cells (MCs) was evaluated with the ethidium (Etd) uptake technique. **(A)** Time-lapse of Etd uptake during 5 min under basal conditions followed by 10 μM OVA stimulation in sensitized WT (black) and Panx1^−/−^ MCs (white). **(B)** Time-lapse measurement of fluorescence intensity of MCs as described in **(A)**. **(C)** Normalized Etd uptake rate values were calculated by determining the fluorescence intensity slope and normalized to the basal conditions. The gray bar represents the Etd uptake rate of non-sensitized control MCs (Ctrl). The first black bar corresponds to the normalized Etd uptake rate during the first 2.5 min of OVA stimulation and the second black bar corresponds to the normalized Etd uptake rate after the first 2.5 min of recording in sensitized WT MCs treated with OVA. All other conditions correspond to sensitized WT and Panx1^−/−^ MCs pretreated during 20 min with a Cx HC blocker [200 μM lanthanum ion (La^3+^) or 200 μM Gap26 to block Cx43 HCs or 200 μM ^10^Panx1 to block Panx1 Chs] followed by OVA stimulation for additional 10 min. In cells treated with La^3+^ or Gap26, the Etd uptake rate was evaluated during the first 2.5 min of stimulation with OVA. Under all other conditions, the dye uptake curve was linear. Each point corresponds to the mean ± SEM, *n* = 3, between 30 and 50 cells were recorded in each experiment, ***p < 0.0005; no significance (n.s.) *p* > 0.05.

Since we previously reported that BMMCs express Panx1 and not Panx2 or Panx3 (24), we decided to evaluate if Panx1 forms functional channels that could increase the cell membrane permeability after cross-linking recognition. To evaluate this possibility, sensitized MCs were placed in a recording solution containing Etd, and after 15 min of stimulation, we found that 10 μM OVA induced dye uptake only in WT but not in Panx1^−/−^ MCs (Figure 3A). In time-lapse experiments, acute OVA exposure rapidly promoted Etd uptake in sensitized WT MCs (Figure 3B). From these experiments, we calculated the changes of fluorescence intensity over time, or dye uptake rate, and normalized the slopes between basal and stimulated conditions. During the first 2.5 min after stimulation with OVA, the normalized Etd uptake rate rapidly and significantly increased [~3.2 times with respect to control conditions (*n* = 6), ^***^p < 0.0005] and then returned to about the basal value [~1.5-fold with respect to control condition (*n* = 6)] (Figure 3C). This increase was prevented with the mimetic peptide ^10^Panx1 (200 μM) and was not detected in MCs from Panx1^−/−^ mice [1.0-fold with respect to control in WT MCs and 1.2-fold in Panx1^−/−^ MCs (*n* = 3), n.s. *p* > 0.05], suggesting strongly the involvement of functional Panx1 Chs found in the cell membrane. The transient increase in Etd uptake suggests the involvement of a regulatory mechanism (e.g., high extracellular ATP concentration) that spontaneously reduces the dye uptake. In addition, a purinergic P2 receptor inhibitor, suramin (100 μM), also prevented the OVA-induced dye uptake in WT MCs (1.2 times with respect to control conditions, *n* = 3). Although it has been reported that MCs also express connexins in their cell membrane (29) that could potentially form membrane HCs permeable to Etd, we discarded their possible contribution during antigen recognition since treatment with connexin HC blockers, such as lanthanum ions (La^3+^) and peptide Gap26, was without effect. We found that La^3+^ (three times with respect to control conditions, *n* = 3) and Gap26 (three times, *n* = 3) did not prevent the increase of Etd uptake observed under these conditions (Figure 3C). The exposure to OVA did not increase significantly the normalized Etd uptake rate in non-sensitized MCs (Figure 3C), suggesting that IgE–FcεRI interaction plays a critical role in OVA-induced activation of Panx1 Chs expressed by MCs.

### Direct ATP Stimulation Restores Panx1-Mediated Cell Responses

**Figure 4:**
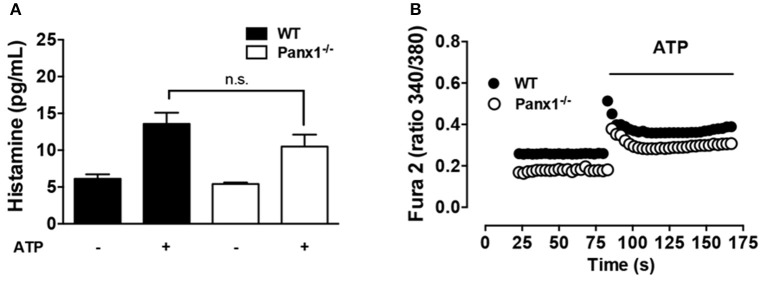
The degranulation and Ca^2+^ signal response of Panx1^−/−^ mast cells are bypassed by exogenous ATP. **(A)** Extracellular histamine concentration in IgE-sensitized WT (black) and Panx1^−/−^ mast cells (MCs) (white) under basal conditions and after 20 min stimulation with 500 μM ATP. **(B)** Intracellular Ca^2+^ signal evaluated with Fura-2 in WT (black) and Panx1^−/−^ (white) MCs. Each value corresponds to the mean ± SEM, *n* = 5; between 30 and 50 cells were recorded in each experiment, no significance (n.s.) *p* > 0.05.

On inflammatory cells, activation of Panx1 Chs is a well-known mechanism for cellular nucleotide release, particularly of ATP (30). In this context, exogenous ATP applied to Panx1^−/−^ MCs could bypass Panx1 Ch-mediated activation upon antigen recognition. Under basal conditions, the extracellular histamine concentration measured in WT and Panx1^−/−^ MCs was similar (Figure 4A). Similarly, after 20 min of 500 μM ATP stimulation, histamine release by Panx1^−/−^ MCs [ATP stimulated Panx1^−/−^ MCs: 10.5 ± 1.6 pg vs. control conditions: 5.4 ± 0.2 pg (*n* = 5); n.s. *p* < 0.05.] was not statistically different to that released by WT MCs [ATP stimulated WT MCs: 13.6 ± 1.5 pg vs. control conditions: 6.1 ± 0.6 pg (*n* = 5); n.s *p* < 0.05] (Figure 4A). Furthermore, resting Ca^2+^ signal was slightly higher in WT compared to Panx1^−/−^ MCs and, after acute stimulation with exogenous ATP, the sustained increase of Ca^2+^ signal that follows the rapid and transient phase after stimulation in Panx1^−/−^ MCs was restored (Figure 4B).

### Panx1 Chs Are Not Permeable to Ca^2+^

**Figure 5:**
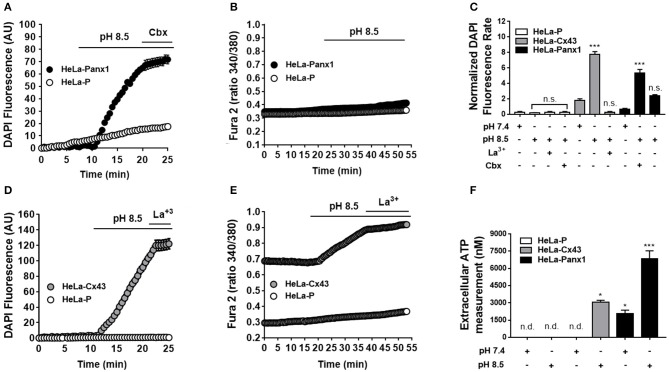
Dye uptake but not Ca^2+^ signal is increased by extracellular alkaline pH via Panx1 Chs. HeLa-Parental cells (HeLa-P) and HeLa cells transfected with Panx1 or Cx43 (HeLa-Panx1 and HeLa-Cx43 cells, respectively) were used to evaluate fluorescence intensity of DAPI (5 μM) uptake (in arbitrary units: AU) and Ca^2+^ signal (calcium imaging of Fura-2-loaded cells, ratio 340/380 nm) was evaluated under different conditions. HeLa-Cx43 were used as positive controls. Opening of Panx1 or Cx43 channels was induced by exposure to alkaline (pH 8.5) solution. During the last 5 min of recording, cells were treated with 5 μM carbenoxolone (Cbx) or 200 μM lanthanum ions (La^3+^) to inhibit Panx1 Chs or Cx43 HCs, respectively. Graphs **(A,C)** show representative time-lapse experiments of dye uptake. Graphs **(B,D)** show representative experiments of Ca^2+^ signal over time under basal conditions and after exposure to extracellular alkaline solution (pH 8.5), in HeLa Cx43 and Panx1, respectively. Each plotted value represents the mean ± SEM. (between 30 and 50 cells were recorded in each experiment). **(E)** Quantification of normalized DAPI fluorescence rate (AU/min) in HeLa parental (white bars, *n* = 3), -Cx43 (gray bars, *n* = 5), and Panx1 cells (black bars, *n* = 5) at pH 7.4 and pH 8.5 bathing solution. **(F)** Measurement of ATP content in the supernatant of HeLa parental (white bars, *n* = 3), -Cx43 (gray bars, *n* = 3), and Panx1 cells (black bars, *n* = 9) after 10 min of control and pH 8.5 bathing solution. In some cases, signal was not detectable (n.d.). ***p < 0.0005; *p < 0.05; no significance (n.s.) *p* > 0.05.

It has been suggested that Panx1 Chs located in the endoplasmic reticulum might be permeable to calcium ions (Ca^2+^) (31). However, to our knowledge, there is no direct demonstration on whether Ca^2+^ can permeate Panx1 Chs found at the cell membrane. To study this possibility without the participation of other Ca^2+^-permeable channels, we used HeLa cells transfected with mouse Panx1. Opening of Panx1 Chs was induced by extracellular alkaline pH, as described previously (32, 33). Upon stimulation, the dye uptake of HeLa-Panx1 cells was increased [from 0.69 ± 0.08 to 5.33 ± 0.39 (*n* = 5), ^***^p < 0.0005] and inhibited by acute treatment with 5 μM carbenoxolone [to 2.41 ± 0.11 (*n* = 5), n.s. *p* > 0.05], a Panx1 Ch blocker (34) (Figures 5A,E). However, in HeLa-Parental cells, used as a negative control as they do not express other Ca^2+^-permeable channels (34), the alkaline stimulation did not affect dye uptake [from 0.28 ± 0.10 to 0.17 ± 0.04 (*n* = 3), respectively] (Figures 5A,E), whereas in HeLa-Panx1 cells, the exposure to extracellular pH 8.5 increased the dye uptake but did not affect the Ca^2+^ signal (Figure 5B). HeLa 43 cells elicited slightly higher basal Ca^2+^ signal compared to HeLa-Parental cells and showed increase in both dye uptake [from 1.78 ± 0.22 to 7.79 ± 0.34 (*n* = 28), ^***^p **<** 0.0005] and Ca^2+^ signal upon exposure to an alkaline solution (Figures 5C,D), a condition known to increase the open probability of these HCs (35). Additionally, quantification of extracellular ATP from supernatants of HeLa transfected cells consistently showed that HeLa Panx1 cells released more ATP upon exposure to alkaline solution [from 2047 ± 318 to 6851 ± 680 nM (*n* = 9), ^***^p **<** 0.0005]. Similarly HeLa Cx43 cells also release more ATP in response to alkalization [from non-detectable signal to 3071 ± 153 (n = 3)], while no ATP was detected in supernatants from parental HeLa cells (Figure 5F). Altogether, these data indicate that open Panx1 Chs do not allow a significant influx of Ca^2+^.

As previously demonstrated (35), HeLa-Cx43 cells exposed to pH 8.5 showed Ca^2+^ influx (35). In addition, we now demonstrated that these open Cx43 HCs enable DAPI uptake and ATP release.

## Discussion

In the present work, we demonstrate that Panx1 Chs play a critical role in the degranulation response of MCs during Type I hypersensitivity reaction promoted by OVA recognition. The absence of Panx1 Chs prevented the development of several MC responses likely to precede the degranulation. Sensitized MCs lacking functional Panx1 Chs did not elicit histamine release or sustained Ca^2+^ signal increase, with both responses being bypassed by exogenous ATP. Moreover, and since Panx1 Chs were not permeable to Ca^2+^, we propose that Panx1 Chs indirectly contribute to the increase in cell membrane permeability to Ca^2+^ because open Panx1 Chs enables the release of ATP. The extracellular ATP can activate P2X receptors permeable to Ca^2+^, which in turn can trigger intracellular events that contribute to the degranulation response.

The biochemical events promoted after cross-linking include the phosphorylation and activation of different tyrosine protein kinases that activate phospholipase C (PLC). Then, PLC hydrolyzes phosphatidylinositol-4,5-bisphosphate (PIP2) generating diacylglycerol (DAG) and inositol-1,4,5-trisphosphate (IP_3_). IP_3_ induces a rapid release of Ca^2+^ from the endoplasmic reticulum (ER), increasing the intracellular free Ca^2+^ concentration (15). The transient rise in IP_3_-induced increase in [Ca^2+^]_i_ is rapidly buffered mainly by mitochondria located close to the ER, which together with the mechanism of extrusion and storage in calciosomes allows the recovering of [Ca^2+^]_i_ to basal values, but reduces the Ca^2+^ content in the ER. It has been proposed that such ER Ca^2+^ depletion promotes a second and persistent increment in [Ca^2+^]_i_. caused by a raise in membrane permeability to this divalent cation (called capacitive Ca^2+^ influx), which would induce the degranulation response (14). As part of the mechanism, it has been proposed that a Ca^2+^ concentration reduction in ER is detected by STIM1, which is translocated from the ER to the cell membrane where it can interact with Orai1 membrane channels. These channels could explain the rapid and transient increase in Ca^2+^ signal recorded in sensitized MCs exposed to OVA. However, the Orai1 channel has low conductance (~0.02 pS), is highly selective for Ca^2+^, and is impermeable for other divalent cations such as Sr^2+^ and Ba^2+^ (36, 37). However, the membrane of activated MCs presents elevated permeability to Ca^2+^, Sr^2+^, Ba^2+^, and Mn^2+^ (38), which cannot be explained only by activated Orai1 channels, suggesting in this way the involvement of additional molecular entities. In agreement with this possibility, we found that the cell membrane of sensitized MCs exposed to OVA become permeable to Etd, which was drastically prevented by ^10^Panx1 peptide (a selective Panx1 Ch blocker) instead of La^3+^ or Gap26, two Cx43 HC blockers (34). Moreover, the increase in membrane permeability to Etd was absent in sensitized Panx1^−/−^ MCs exposed to OVA, unraveling a critical role for Panx1 Chs in the OVA-induced membrane permeability of antigen sensitized MCs.

Relevant questions could be the following: How are Panx1 Chs activated in sensitized MCs exposed to OVA? Could Panx1 Chs be activated by the rapid and transient increase in Ca^2+^ signal generated after antigen recognition? Orai1 channels might play a relevant role leading to Panx1 Chs activation for they are activated by an increase in intracellular Ca^2+^ concentration (19). Accordingly, in sensitized Panx1^−/−^ MCs, OVA only induced a rapid and transient increase in Ca^2+^ signal possibly via a mechanism that involves the activation of PLC and generation of IP_3_ that induces Ca^2+^ release from intracellular stores as already mentioned. In this context, and since Panx1 does not contain Ca^2+^ binding sites, Panx1 Ch activation mechanism might be through a Ca^2+^-dependent protein phosphorylation (38). Accordingly, Panx1 has two threonine residues (Thr302 and Thr384) in consensus sites for calmodulin-dependent protein kinase II (38). Nevertheless, further studies are required to determine their possible participation in activation of Panx1 Chs. Interestingly, we found that the increase in membrane permeability to Etd via Panx1 Chs was transient (~2.5 min), which might be explained by inhibition of Panx1 Chs by a high extracellular ATP concentration due to opening of the same Panx1 Chs (19).

Consistent with the notion that Panx1 Chs are non-selective membrane channels, we found that exposure to extracellular alkaline solution, known to promote opening of fish Panx1 isoforms (32, 33), also induces opening of mammalian Panx1 Chs. The latter was revealed by the increase of ATP release and DAPI uptake inhibited by low (5 μM) carbenoxolone concentration that acts as Panx1 Ch blocker (34). However, in HeLa Panx1 cells, pH 8.5 did not affect the Ca^2+^ signal, suggesting that these channels did not allow Ca^2+^ influx in MCs.

Considering the fact that the alkaline solution could activates other Ca^2+^ permeable membrane channels (e.g., through ACIS, Piezol or TRP channels), we cannot rule out the possible involvement of other membrane channels activated by alkaline pH that could indirectly leads to activation of Panx1 Chs. However, in HeLa-Parental cells, we could not detect an increase in membrane permeability to DAPI or ATP release in response to alkaline pH, suggesting that these responses of HeLa transfectants are in fact mediated by Panx1 Chs and therefore indicate that Panx1 Chs are directly activated by alkaline pH.

In support to the above proposal, it is conceivable that P2X receptors activated by extracellular ATP released via Panx1 Chs participates in the plateau phase of the Ca^2+^ signal of sensitized MCs exposed to OVA. Accordingly, several P2X receptors are permeable to Ca^2+^ (38) and their involvement in the plateau phase of the Ca^2+^ signal is supported by the following findings: (1) the dye uptake response was promoted by 500 μM ATP, which is a concentration that activates P2X receptors (38). (2) The dye uptake induced by OVA was prevented by suramin, which blocks P2 receptors (38). (3) In Panx1^−/−^ MCs, exogenous ATP bypassed the absence of Panx1, promoting histamine secretion and Ca^2+^ signal similar to that induced by OVA in WT MCs. As additional support, it is relevant to notice that P2X receptors do not desensitize upon activation by high extracellular ATP concentration (38), and in this way, their persistent open state can explain the long-lasting duration (>80 s) of the plateau phase of the Ca^2+^ signal. Accordingly, it has been shown that MCs express a wide variety of damage-associated receptors involved in Type I hypersensitivity reactions, like purinergic P2X receptors (39).

**Figure 6:**
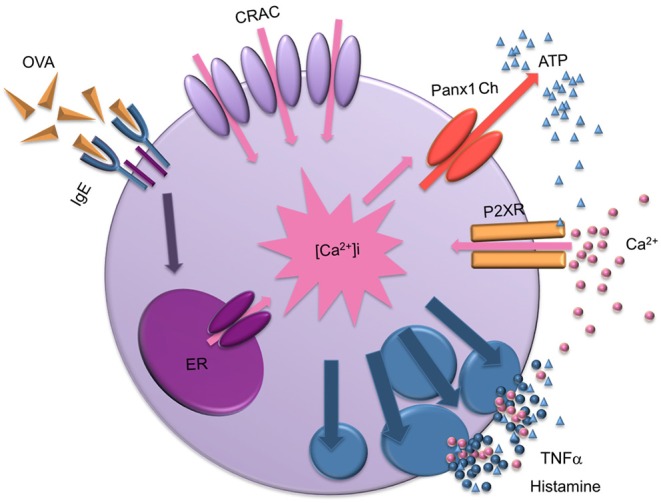
Scheme of the mast cell degranulation process induced by IgE cross-linking and the involvement of Panx1 channels. After antigen recognition, biochemical events lead to Ca^2+^ release from intracellular stores likely to be the endoplasmic reticulum (ER), causing a transient increase in cytoplasmic Ca^2+^ concentration ([Ca^2+^]_i_). Then, Ca^2+^ released from the ER activates the Ca^2+^ influx through membrane channels (calcium released activates CRAC channels) slowly replenishing intracellular stores. Sustained increase in Ca^2+^ signal also favors the transient opening of Panx1 Chs, allowing the release of ATP to the extracellular media and activation of purinergic receptors (P2XRs). Active P2XRs contribute to the sustained increase of [Ca^2+^]_i_ , promoting activation of Panx1 Chs and mast cell degranulation, leading to release of pro-inflammatory compound such as TNF-α and histamine. Finally, high extracellular ATP concentration could block Panx1 Chs.

In summary, after OVA cross-linking recognition, characterized by (1) a rapid and transient Ca^2+^ signal, (2) followed by a sustained Ca^2+^ influx, we propose that Panx1 Chs indirectly contribute to the latter event, since they are not permeable to Ca^2+^. In this context, Panx1 Chs opening leads to activation of a purinergic signal pathway through an autocrine process, as ATP released through Panx1 Chs could activate P2X receptors. These key events lead to degranulation and consequent histamine release to the extracellular milieu, a characteristic feature of Type I hypersensitivity reactions (Figure 6).

## Data Availability Statement

The datasets generated for this study are available on request to the corresponding author.

## Ethics Statement

The animal study was reviewed and approved by Comisión de Bioética y Bioseguridad de la Pontificia Universidad Católica de Chile.

## Author Contributions

PH and JS designed the experiments and wrote the paper. PH, XL, PS, PF, IB, and AM performed the experiments. PH analyzed the data.

### Conflict of Interest

The authors declare that the research was conducted in the absence of any commercial or financial relationships that could be construed as a potential conflict of interest.
